# Understanding the CD8 T-cell response in natural HIV control

**DOI:** 10.12688/f1000research.15029.1

**Published:** 2018-07-02

**Authors:** Sushma Boppana, Paul Goepfert

**Affiliations:** 1 Department of Medicine, University of Alabama at Birmingham, Birmingham, USA

**Keywords:** HIV Controllers, CD8 T cells

## Abstract

HIV-infected individuals who maintain control of virus without antiretroviral therapy (ART) are called HIV controllers. The immune responses of these individuals suppress HIV viral replication to low levels or, in the case of elite controllers, to undetectable levels. Although some research indicates a role for inferior virulence of the infecting viral strain in natural control, perhaps by way of defective Nef protein function, we find that the majority of research in HIV controllers highlights CD8 T cells as the main suppressor of viral replication. The most convincing evidence for this argument lies in the strong correlation between certain HLA-I alleles, especially B*57, and HIV control status, a finding that has been replicated by many groups. However, natural control can also occur in individuals lacking these specific HLA alleles, and our understanding of what constitutes an effective CD8 T-cell response remains an incomplete picture. Recent research has broadened our understanding of natural HIV control by illustrating the interactions between different immune cells, including innate immune effectors and antigen-presenting cells. For many years, the immune responses of the natural HIV controllers have been studied for clues on how to achieve functional cure in the rest of the HIV-infected population. The goal of a future functional cure to HIV is one where HIV-infected individuals’ immune responses are able to suppress virus long-term without requiring ART. This review highlights recent advances in our understanding of how HIV controllers’ natural immune responses are able to suppress virus.

## Introduction

This review aims to summarize the field’s current understanding of natural HIV controllers, with a focus on recent advances examining the CD8 T-cell response in these individuals and harnessing this knowledge for future therapies. A difficulty with comparing different studies on HIV controllers is the variation in definitions that different groups use, as illustrated by Gurdasani *et al*.’s systemic review of 714 definitions in 501 elite controller studies ^1^. For this review, we will assume the following definitions and specify any deviations: HIV controllers maintain viral loads of less than 2,000 RNA copies per milliliter (mL) of plasma off-antiretroviral therapy (ART). This group of HIV controllers can be further divided into elite controllers, individuals who maintain undetectable viremia (HIV RNA <50 copies/mL), and viremic controllers, who maintain low, but detectable, viremia (HIV RNA <2,000 copies/mL). Some studies focus on long-term non-progressors (LTNPs), who are HIV-infected individuals who remain asymptomatic for a prolonged period time off-ART with CD4 cell counts higher than 500, and do not restrict based on viral load.

A major question in the field asks what the relative contribution of viral functions and host factors are to natural HIV control. Although some cases demonstrate that infection with less-virulent HIV strains allows for better natural control, based on the evidence available, the host immune response, especially the CD8 T-cell response, appears to more strongly contribute to natural control.

## Viral functions contributing to control

Several studies indicate that the function of Nef, the negative factor protein encoded by HIV, is impaired in HIV controllers. Typically, Nef is responsible for down-regulating several surface markers, including CD4, on HIV-infected cells and helps perpetuate the infection. Several groups first described the connection between infection with a Nef-deleted or -defective virus and natural viral control in 1995 ^2, 3^. However, more recent studies of Nef function in elite controllers show that while individuals can be infected with a Nef-defective virus, these less-virulent viruses are often a result of the controllers’ CD8 T cells exerting immune pressure and causing an accumulation of HLA-I-associated polymorphisms within the Nef viral sequence. This accumulation of CD8 escape mutations then results in impaired Nef functionality ^4, 5^, perhaps by increasing the sensitivity of infected cells to antibody-dependent cellular cytotoxicity (ADCC) ^6, 7^.

There is also some evidence indicating that viruses isolated from elite controllers have a poorer replicative capacity than those from non-controllers. Investigations of Gag and Pol sequences indicate that HIV in controllers is less replication competent; however, like Nef impairment, this weakened replicative fitness is caused by fitness-costly escape mutations, a result of the host CD8 T-cell response ^8, 9^. A recent study of the Env sequences showed that viruses isolated from elite controllers had impaired CD4 binding and signaling compared to non-controllers and asserts virologic factors play a role in HIV control in these individuals ^10^. Other studies have described instances where the same virus caused significant pathogenicity in some HIV-infected individuals but was effectively suppressed by the CD8 T-cell response in HIV controllers ^11– 13^. Similarly, characterization of the viral fitness and early immune response in a recently infected controller demonstrated that the individual maintained viral control even with a replication-competent virus through a potent CD8 T-cell response that prevented escape mutations from occurring in the Gag protein ^14^. Another recent study examining superinfection in a B*57-positive progressor showed that disease progression occurred in spite of significant decreases in viral fitness, indicating that decreased viral fitness is not sufficient for maintained viral control ^15^.

## CD8 response in HIV controllers

**Figure 1.**
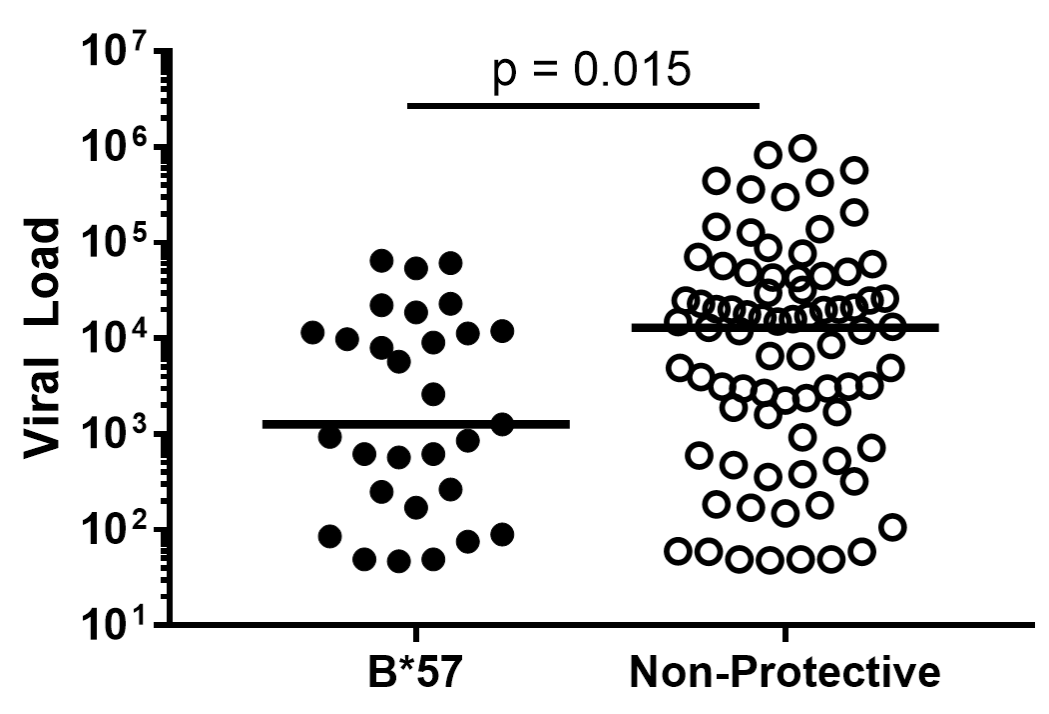
Association of B*57 with viral control in 1917 clinic cohort. Viral loads of 1917 clinic patients with protective HLA-B*57 allele versus those of patients lacking any HLA-I alleles previously associated with delayed or accelerated disease progression.

Perhaps the strongest evidence that the host CD8 T-cell response is the major player in natural control of HIV is the association of specific HLA-I alleles, like B*27 and B*57, with elite controllers; this finding is consistent across several groups ^16– 19^. HLA-I alleles dictate which epitopes from HIV are presented to and recognized by the host immune response, and these protective HLA-I alleles drive potent and effective CD8 T-cell responses ^20^. In fact, this relationship is so strong that it can be seen even within a single clinic’s cohort. In the University of Alabama at Birmingham’s HIV clinic, 1917 clinic, B*57-positive HIV-infected patients are more likely to be controllers ( *P* = 0.04, Fisher’s Exact test) and have lower viral loads ( *P* = 0.015 by Mann Whitney test, Figure 1). Carlson and colleagues recently demonstrated the importance of CD8 T cells in viral control by examining the relationship between the “pre-adaptation” of the infecting virus and subsequent control of that virus. They found that individuals infected by a virus already containing CD8 T-cell escape mutations had poorer clinical outcomes, including higher viral load set points and faster CD4 T cell decline. Importantly, the advantage conferred by protective HLA-I alleles, like B*57, was lost when B*57-positive individuals were infected with pre-adapted virus, indicating that viral control in elite controllers is dependent on the CD8 T-cell response’s ability to prevent CD8 escape mutations from accumulating ^21^.

The CD8 T-cell response of HIV controllers is more robust than that of non-controllers in several ways, displaying greater cytotoxicity ^22, 23^, proliferation ^24, 25^, and polyfunctionality ^26– 28^. However, our understanding of the CD8 T-cell response in HIV controllers is continuing to evolve. For example, a more recent study found that freshly thawed cells from controllers and progressors were similarly able to inhibit virus but that CD8 T cells from controllers exhibited better sustained polyfunctionality over time ^29^. Two recent studies transfected cells with the T-cell receptors (TCRs) from an elite controller’s CD8 T-cell response and found that the superior functionality of CD8 T cells is not completely dictated by the TCR itself and that factors external to the TCR sequence contribute to antiviral functionality ^30, 31^. Although many aspects of the CD8 T-cell response are superior in controllers, whether these CD8 T-cell characteristics are the cause of greater viral control or the result of a more preserved immune response because of low or absent viremia often remains uncertain.

Another beneficial aspect of the CD8 T-cell response of elite controllers is thought to be early and potent targeting of the Gag protein, a key structural and functional protein of HIV. Brockman *et al*. found that viruses from individuals with protective alleles had lower replication capacity, which inversely correlated with HLA-B-associated Gag polymorphisms in early HIV infection. This correlation suggests that the early CD8 T-cell response in these individuals created immune pressure and forced the virus to undergo fitness-costly mutations within Gag ^32^. Additionally, the magnitude of the anti-Gag CD8 T-cell response correlates with the level of viral inhibitory activity ^33^. These potent CD8 T-cell responses are seen in elite controllers with and without protective HLA-I alleles ^34^.

The CD8 T-cell responses of elite controllers may also be more cross-reactive than those of HIV progressors, meaning their CD8 T cells may be better equipped to respond to not just the epitopes encoded by the infecting virus but also variant epitopes that arise as a result of HIV’s rapid accumulation of mutations. One study computationally demonstrated that protective HLA-I alleles, like B*57, present fewer self-peptides in the thymus, resulting in a T-cell repertoire that relies on a lower number of residues to recognize viral peptides and, therefore, a T-cell response that is able to recognize a much broader array of variant epitopes ^35^. This increased cross-recognition was also observed in *ex vivo* and *in vitro* studies where higher cross-recognition scores were associated with lower viral loads ^36^ and T-cell clones derived from controllers were more cross-reactive than clones derived from HLA-matched progressors ^37^. CD8 T cells from B*57-positive elite controllers have been found to effectively suppress the replication of viruses encoding escape mutations, demonstrating an ability to suppress variant epitope-encoding viruses ^38^.

Some HIV controllers eventually lose their ability to suppress virus without therapy, and investigations into the mechanisms behind this loss of control may provide clues into which actors exert control in the first place. A recent study comparing elite controllers with and without CD4 decline found that disturbances in T-cell homeostasis, including lower levels of naïve CD8 T cells, fewer recent thymic emigrants, and greater CD4 T-cell exhaustion, appeared to lead to this loss of control and rapid CD4 T-cell decline ^39^. Most of the disturbances in homeostasis that correlated with CD4 T-cell loss in this study involved the CD8 T-cell response, supporting the idea that CD8 T cells are the major player in the viral control existing before CD4 loss occurred.

## Parallels in model systems

Some non-human primate (NHP) models are seen as comparable to natural HIV controllers. Simian immunodeficiency viruses (SIVs), which are related to HIV, replicate efficiently in their natural hosts (e.g. African green monkeys and sooty mangabeys) without causing any disease pathology, but the SIV/macaque model, which is often used for HIV studies, involves laboratory-selected SIV strains, which are pathogenic. Watkins and colleagues have linked HIV control in the presence of the protective HLA-I allele B*27 with Mamu B*08-mediated SIV control in rhesus macaques where roughly 50% of Mamu B*08-positive macaques control SIV replication similar to elite controllers ^40^. They have also demonstrated that an effective CD8 T-cell response in acute infection is key and that early CD8 T-cell-mediated viral escape occurs in Mamu B*08 progressors but not in Mamu B*08 controllers ^41^. Additionally, upon depletion of CD8 T cells in controller monkeys, researchers observed a major rebound in virus, even greater than that seen in progressors ^42, 43^. These studies of rhesus macaques suggest that the CD8 T-cell response is the primary effector in natural SIV control. Another recently published study lends support to this idea that viral factors are not the key influencers of disease progression in NHP controllers. Joas *et al*. examined the impact of adding or replacing pathogenic HIV-encoded Vpu and Nef, two important HIV virulence factors, to a non-pathogenic SIV strain. They found that despite high levels of viral replication, Vpu- and/or Nef-containing SIV strains remained non-pathogenic. Because the introduction of these virulence factors did not increase the pathogenicity of the tested SIV strains, the authors concluded that host factors are more likely to be responsible for protection against disease ^44^. Overall, these studies in NHP models of SIV infection lend strong support to the importance of CD8 T cells in natural control.

## Other host functions contributing to control

Recent studies have also investigated the contribution of the innate immune system to natural HIV control. Krishnan and colleagues found that D-dimer, soluble tissue factor, and CD14 ^+^CD16 ^+^ monocyte levels were elevated in elite controllers, indicating a more activated innate immune response ^45^. Another recent study looked at differences in systemic cytokine responses between elite controllers and non-controllers and found that a subset of cytokines was increased in controllers and that this combination of cytokines (CCL14, CCL21, CCL27, XCL1, and SDF-1) was able to suppress viral replication *in vitro* ^46^. Additional research is required before we fully understand the complex roles of innate immune actors on viral control in HIV controllers.

The antigen-presenting cells (APCs) are also an important component of the immune response, and their role in HIV control is becoming clearer. Martin-Gayo and colleagues used a computational framework and single-cell RNA sequencing to describe a higher functional antiviral dendritic cell state that primes polyfunctional T-cell responses in elite controllers ^47^. In addition to aiding the CD8 response, dendritic cells and other APCs may contribute to natural control through an innate resistance to spreading virus from cell to cell, or trans-infection. For dendritic cells, B lymphocytes, and macrophages, the Rinaldo group demonstrated that this decreased facilitation of trans-infection is linked to altered cholesterol metabolism. In HIV non-progressors, they found that APCs express less free cholesterol on the cell surface, leading to a decreased internalization of virus ^48, 49^. To date, APCs in HIV have been understudied, and future research will likely highlight the important ways in which these cells interact with effector immune cells.

Recent research has also noted significant differences between male and female HIV controllers (reviewed in 50); women are overrepresented among HIV controllers in several studies ^51, 52^. Zhang *et al*. found that female elite controllers actually had similar gene expression profiles to uninfected females but differences from male elite controllers. Because the differentially expressed genes between men and women in the study were not exclusively located on the X or Y chromosome, the authors argue that these gene expression differences are not simply due to sex-linked genes ^53^. Another study indicates that significantly fewer female elite controllers experience loss of natural HIV control as defined by CD4 T-cell decline ^39^. In fact, even within the 1917 clinic, we see that women are significantly overrepresented within the elite controller population compared to the general clinic population ( Table 1, *P* = 0.03). Recent publications indicate a need for more research into sex differences in HIV controllers and a better understanding of how these differences might play into future therapeutic efforts.

**Table 1.** 1917 clinic demographics, female versus male elite controllers.

	Females		Males		Total
	Number	Percent	Number	Percent	Number
Active patients	839	24%	2,618	76%	3,457
Elite controllers	9	47%	10	53%	19

Active patients defined as being seen in the 1917 clinic within the past year; elite controllers defined as individuals with viral loads of <50 copies of RNA/mL of plasma.

The CD4 response may also play an important role in natural HIV control. A recent study of the TCRs responsible for the immunodominant CD4 response in controllers found a significant number of public clonotypes, including shared CDR3 motifs. Transfer of these Gag-specific, high-avidity TCRs to non-HIV-infected CD4 T cells resulted in high antigen sensitivity and polyfunctionality and even redirected CD8 T cells to target the HIV capsid ^54^. A follow-up study found that these CD4 TCRs were able to suppress HIV in the context of multiple genetic backgrounds, raising the possibility of engineering or transferring CD4 T cells as an effective therapy ^55^. Although CD4 T cells may play a supportive role in controlling HIV in these individuals, depletion of CD4 T cells in SIV-infected macaque elite controllers does not impact viral load, indicating that they are not required for natural control ^56^.

## Future directions

A current area of interest for the HIV cure field is the dynamics of the latent HIV reservoir within infected individuals because this reservoir poses a major barrier to achieving HIV cure. Boritz and colleagues studied the persistence of virus in HIV controllers and found that, despite effective antiviral immune responses in these individuals, their reservoir was maintained through viral replication, clonal expansion of latently infected cells, and a low level of circulating infected cells ^57^. However, it was recently demonstrated that the HIV reservoir is smaller in elite controllers, particularly in peripheral T follicular helper cells, meaning the host response within these controllers is able to limit the HIV reservoir size ^58^. The HIV reservoir found in lymphoid tissue is a major barrier to current CD8 T-cell-based cure efforts because without effective immune responses or daily ART, these latently infected cells reactivate and virus rebounds. Further research into how natural HIV controllers are able to suppress the emergence of virus from these pockets could be a useful step forward.

A caveat to using natural HIV control to inform functional cure strategies is recent research indicating that in spite of long-term ART-free viral control, elite controllers may still have increased levels of inflammation compared to HIV-negative individuals. Even in HIV controllers, viral evolution can be seen, indicating that these individuals have some level of ongoing viral replication ^59, 60^. This low-level persistence of HIV in controllers may contribute to the described increased levels of inflammatory markers, like IFNγ, IL10, and sCD40L ^61^. However, another publication from the same year found that elite controllers with preserved CD4 T-cell percentages do not have increased levels of T-cell activation ^62^, and more recent research in *JAMA Cardiology* found that arterial and lymph node inflammation in elite controllers was comparable to that of ART-suppressed HIV-infected individuals ^63^. Additionally, analysis of non-AIDS-defining events (nADEs) found that while controllers do experience nADEs, they do so at a lower rate than do non-controllers ^64^. Further research into the potential long-term effects of natural HIV control is required to confirm that natural control is the ideal model for developing a functional cure for HIV.

There is still hope that our understanding of natural control can be used to inform future vaccines or therapy strategies. Although we have made significant progress in understanding the dynamics underlying the CD8 T-cell responses in these individuals, the field has successfully harnessed effective CD8 T-cell responses to counter HIV infection only in the lab and in macaque models. While antibodies are likely to play a major role in preventing HIV infection, preventative vaccines that pack a powerful CD8 arm may be able to alleviate the course of disease in vaccinated individuals who become infected. Based on our understanding of natural controllers’ CD8 responses, this vaccine-induced response would likely need to effectively prevent the virus from escaping the immune response or would need to force more fitness-costly mutations to occur. CD8 T cells restricted by HLA-E and possibly HLA-II could also be harnessed to prevent establishment of HIV infection, as has been elegantly demonstrated by the Picker group using cytomegalovirus recombinant SIV vectors in the NHP model system ^65, 66^. CD8 T cells may also be the central effector of future functional cures, where the CD8 T-cell response of an infected individual is reinvigorated and redirected to effectively suppress virus in the absence of ART. Future research on natural HIV control can help the field move closer to achieving these goals.

## Abbreviations

ADCC, antibody-dependent cellular cytotoxicity; APC, antigen-presenting cell; ART, antiretroviral therapy; LTNP, long-term non-progressor; mL, milliliter; nADE, non-AIDS-defining event; NHP, non-human primate; SIV, simian immunodeficiency virus; TCR, T-cell receptor
